# Randomized Comparative Study Between Bilateral Erector Spinae Plane Block and Transversus Abdominis Plane Block Under Ultrasound Guidance for Postoperative Analgesia After Total Abdominal Hysterectomy

**DOI:** 10.7759/cureus.25227

**Published:** 2022-05-22

**Authors:** Usha Shukla, Urvashi Yadav, Amit K Singh, Abhishek Tyagi

**Affiliations:** 1 Anaesthesiology and Critical Care, Uttar Pradesh University of Medical Sciences, Etawah, IND; 2 Anaesthesiology, Uttar Pradesh University of Medical Sciences, Etawah, IND

**Keywords:** nrs pain score, postoperative analgesia, erector spinae plane block, transversus abdominis plane block, s: abdominal hysterectomy

## Abstract

Introduction: Ultrasound-guided erector spinae plane (ESP) block has emerged as an effective and safe analgesic regional technique and it also provides visceral pain relief. Our aim was to compare the analgesic efficacy of ESP block over transversus abdominis plane (TAP) block under ultrasound guidance following a total abdominal hysterectomy.

Methods: This was a prospective, randomized, comparative study. Thirty females posted for elective open total abdominal hysterectomy under general anesthesia were randomly allocated into two groups. Ultrasound-guided ESP block was applied in group E at the T-9 level bilaterally. The study solution was prepared by mixing 20 ml of 0.5% bupivacaine plus 10 ml of 2% lignocaine and 1 ml (50mcg) of fentanyl and 9 ml of normal saline forming total 40 ml of which 20 ml was injected on each side. Group T received ultrasound-guided TAP block with 20 ml of study solution bilaterally. The study solution was prepared similarly by mixing 20 ml of 0.5% bupivacaine plus 10 ml of 2% lignocaine and 1 ml (50mcg) of fentanyl and 9 ml of normal saline (total 40 ml) of which 20 ml was injected into each side. Tramadol 100mg iv was given as rescue analgesia whenever NRS ≥ 4 or on-demand in the postoperative period. The primary outcome was changes in a numerical rating scale (NRS) pain score postoperatively between two groups in 24 h, duration of analgesia and total rescue analgesic required during 24 h. The secondary outcome was patient satisfaction level and side effects if any.

Results: Demographic data were comparable in both groups. The NRS pain score was significantly lower ​​​​in group E than in group T at second, third, fourth, fifth (p < 0.001) and at sixth hour (p < 0.05) postoperatively. The mean duration of analgesia was significantly more in Group E (4.73±0.7 h) compared to group T (2.60±0.51 h) (p < 0.001). The tramadol consumption was seen significantly more in 24 h in group T (233.33±25.82 mg) than in group E (193.33±17.59 mg). Patient satisfaction score was significantly higher at 12 h with mean value of 6.07±0.26 in group E compared to 3.40±0.91 in group T.

Conclusion: We conclude that ultrasound-guided ESP block provide better postoperative pain control and prolonged duration of analgesia with less tramadol consumption compared to ultrasound-guided TAP block in patients after total abdominal hysterectomy.

## Introduction

Hysterectomy represents the second most common obstetric surgery after cesarean section. Total abdominal hysterectomy is preferred in patients with enlarged or bulky uterus where the minimally invasive route is considered technically challenging [1]. Poor pain control after major abdominal surgeries is associated with unwanted consequences such as patient discomfort, delayed recovery, and prolonged hospital stay [2]. Administration of systemic drugs such as opioids and NSAIDs, and infiltration of local anesthetic in the skin around the surgical wound have been tried but has many side effects like respiratory depression, sedation, nausea, and vomiting, which delay rehabilitation and early discharge [3,4].

Transversus abdominis plane (TAP) block, has been described by Rafi AN in 2001 [5]. In the TAP block, the intercostal, subcostal, and L1 segmental nerves communicate to form the upper and lower TAP plexuses, which innervate the anterolateral abdominal wall, including the parietal peritoneum [6]. TAP block has been used for a variety of abdominal surgeries. There are several different approaches for ultrasound-guided TAP block, and are categorized into four groups comprising subcostal, oblique subcostal, lateral and posterior approaches. We used lateral approach in our study. The erector spinae plane (ESP) block first described in 2016 is a newer regional anesthetic technique that can be used to provide analgesia for a variety of surgical procedures or to manage acute or chronic pain. Recently USG guided ESP block has been used in many thoracic and abdominal surgeries for control of postoperative pain [7]. There are limited studies comparing these two blocks for postoperative pain [8-12].

We aim to evaluate and compare the analgesic efficacy and duration of analgesia provided by ESP block and TAP block applied under ultrasound guidance.

## Materials and methods

This study was conducted after approval from the Institutional ethical committee of the university(with Ref no:1659/UPUMS/Dean(M)/Ethical/20-21, EC No:74/2019-20 dated 6/4/2021). Informed consent was taken from all 30 patients of the American Society of Anesthesiologist physical status classification I and II, age group 35 to 60 years, body mass index (BMI) 18-32 kg.m^-2^ scheduled for elective open total abdominal hysterectomy. Patients suffering from any medical illness like cardio-pulmonary, hepato-renal, or metabolic disorders or patients with mental problems having difficulty understanding the scoring for pain were excluded from the study. Sample size calculation was done based on a previous study considering 5% alpha error and power of 80% using SPSS (version 20; IBM, Armonk, NY, USA) software. All patients received oral tab. 0.5 mg alprazolam and tab ranitidine 300 mg on the night before surgery. In the operation room, an intravenous (iv) cannula was inserted and iv fluids were started. Baseline vital parameters were noted such as heart rate, noninvasive blood pressure, five lead electrocardiogram, and oxygen saturation.

Sealed envelope randomization was employed to allocate patients into two groups either to receive ESP block (group E) or TAP block (group T). The anesthesiologist who administered the block was not involved in data collection. Group E (n=15) received bilateral ESP Block with 20 ml local anesthetic (LA) solution on each side. LA solution was prepared by adding 20 ml of 0.5% bupivacaine, 0 ml of lignocaine 2%, 1 ml (50 mcg) of fentanyl, and 9 ml of normal saline to make a total volume of 40 ml of which 20 ml was injected on each side.

Group T (n=15) received bilateral TAP Block with 20 ml of LA solution on each side. LA solution was prepared by adding 20 ml of 0.5% bupivacaine, 10 ml of lignocaine 2%, 1 ml (50 mcg) of fentanyl, and 9 ml of normal saline to make a total volume of 40 ml of which 20 ml was injected on each side.

**Figure 1:**
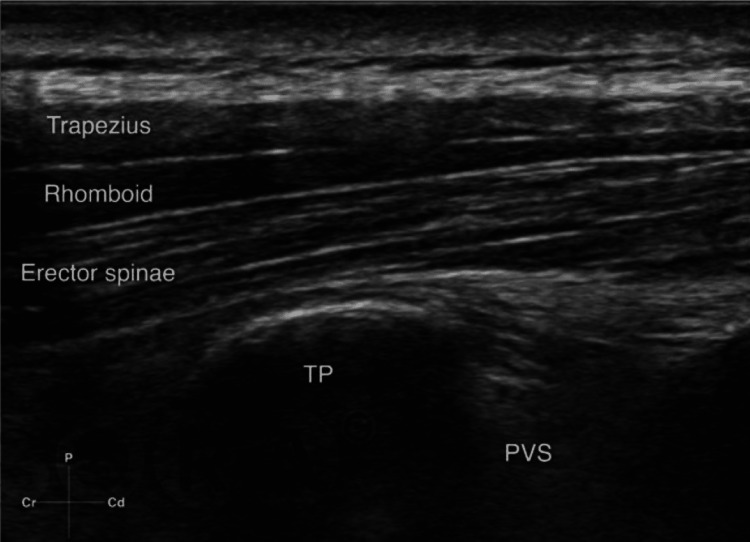
The position of needle in plane superior to inferior approach to ESP block. TP = transverse process, ESP = erector spinae plane

In group E, after assuring skin asepsis, a high frequency (5-13 MHz) linear ultrasound transducer in the sterile sheath was implanted at about 3 cm lateral to thoracic-9 spinous process to observe the back muscles from above to below: trapezius, then rhomboid major, and then erector spinae. Using an in-plane superior to inferior approach, a 23G 10-cm Touhy needle was introduced until it contacted the transverse process (TP), which crossed all three muscles (Figure 1). When the TP was contacted and 1 ml of LA anesthetic solution was administered after confirming the position of the needle under ultrasound in plane view. Further hydro-dissection of the plane was seen when a full 20 ml volume of LA solution was injected. Similarly, a block was applied on the contralateral side also.

**Figure 2:**
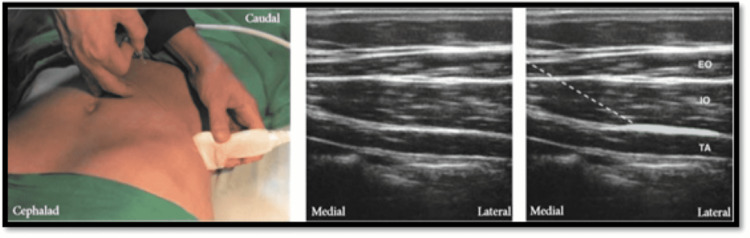
Lateral TAP block approach. EO - external oblique muscle, IO - internal oblique muscle, TA - transversus abdominis muscle.

In group T patients, after ensuring skin asepsis, a high-frequency (5-13 MHz) linear ultrasound transducer in a sterile sheath was placed in between lower costal margin and the iliac crest transversally and the transversus abdominis muscle was identified below the external and internal oblique muscle, 20 ml of LA solution was injected between the fascia immediately above the rectus abdominis muscle (Figure 2). The same procedure was done on other side using lateral TAP block approach.

Inj glycopyrrolate 10 g.kg^-1^ iv, inj midazolam 0.05 mg.kg^-1^ iv, and inj fentanyl 2 g.kg^-1^ iv were given to all of the patients as premedication. Anesthesia was induced with propofol iv (2 mg.kg^-1^) and patients were intubated with vecuronium (0.1 mg.kg^-1^) iv after a three-minute preoxygenation with 100% oxygen. Intraoperative analgesia was provided by inj paracetamol 15mg/kg. Isoflurane 1%-1.5%, 50% nitrous oxide in oxygen, and additional doses of vecuronium were used to maintain anesthesia (0.01 mg.kg^-1^). The neuromuscular block was restored with neostigmine 0.05 mg.kg^-1^ and glycopyrrolate 0.01 mg.kg^-1^ iv at the end of the procedure, and the trachea was extubated once respiration was satisfactory and the patient could obey verbal orders. All of the patients were evaluated for changes in hemodynamic parameters such as blood pressure, heart rate and saturation preoperatively, after block (baseline) followed by every 5 min for 30 min then every 10 min till the end of surgery and 15 min after extubation in post-op. The primary outcomes measured were (1) pain intensity using NRS score immediately and then at 15 min, 30 min, 45 min, 60 min, 90 min, second hour, third hour, fourth hour, fifth hour, sixth hour, 12th hour, and 24th hours postoperative. (2) The time of first analgesic demand (hours); and (3) the total amount of tramadol consumption during the first 24 hours of postoperative period. Secondary outcomes measured were (1) overall patient satisfaction score at the end of surgery and at 12 hours postoperatively and any adverse events such as bradycardia (HR < 50 bpm or 20% decrease from the baseline value), hypotension (fall in blood pressure by 20% from the baseline or an absolute MAP < 60 mmHg), nausea, vomiting, or any other events during and after procedure were also noted. A 7-point Likert verbal rating scale was used to assess patient satisfaction immediately after surgery and at 12 hours after surgery as follows: 1-Extremely Dissatisfied, 2-Dissatisfied, 3-Somewhat Dissatisfied, 4-Undecided, 5-Somewhat satisfied, 6-Satisfied, 7-Extremely satisfied.

Data were analyzed with SPSS version 20. Quantitative data were summarized as mean ± standard deviation and analyzed by an unpaired t-test. The qualitative data was expressed as number and percentage and were analyzed by chi-square test. P-value < 0.05 was considered statistically significant and highly significant if < 0.001.

## Results

All patients participated in the study. No statistically significant differences were found between the groups regarding patient demographic profiles or clinical characteristics. There was no difference in the age of patients and duration of surgery (Table 1). Vital parameters like heart rate, blood pressure, and saturation were comparable during the intraoperative and postoperative period.

**Table 1:** Patient characteristics and duration of surgery. BMI = body mass index, ASA American Society of Anesthesiologist, SD = Standard Deviation.

Characteristics	Group E (n=15) (Mean + SD)	Group T (n=15) (Mean + SD)	P-value
Age (years)	43.60 ± 8.93	46.33 ± 10.67	0.226
Height (cm)	151.7 ± 6.1327	154.4 ± 7.6411	0.1366
BMI (kg/m^2^)	25.166 ± 2.288	26.04 ± 1.561	0.8041
ASA (Ⅰ/Ⅱ)	8/7	9/6	0.297
Duration of surgery (Min)	104.00 ± 14.04	104.67 ± 14.57	0.450

NRS pain scores were significantly lower (p < 0.05) in the second, third, fourth, fifth hour, and sixth hour in group E compared with group T (Table 2). As we can see from the table, patients started complaining of pain 90 min postoperatively but statistically, a significant difference was found between the groups after 120 min. In the T group, the pain started earlier whereas the onset of pain was delayed in the E group.

**Table 2:** Comparison of NRS pain score in both groups. h= hour, * significant, ** highly significant

NRS Score	Group T (n=15)		Group E (n=15)		p-value
	Mean	±SD	Mean	±SD	
Immediate Postop	0.00	±0	0.00	±0	-
15 min	0.00	±0	0.00	±0	-
30 min	0.00	±0	0.00	±0	-
45 min	0.00	±0	0.00	±0	-
60 min	0.00	±0	0.00	±0	-
90 min	0.80	±0.68	0.53	±0.52	0.117
120 min	3.13	±1.13	0.00	±0	<0.001**
3hr	2.87	±2.47	0.60	±0.74	0.001**
4hr	0.00	±0	2.60	±1.55	<0.001**
5 h	0.00	±0	2.60	±2.29	<0.001**
6 h	1.67	±1.18	0.60	±1.4	0.016*
12 h	3.00	±0.53	2.93	±0.26	0.333
24 h	4.47	±0.74	4.40	±0.63	0.397

The duration of analgesia was significantly (p < 0.001) more in E group with mean value of 4.73±0.7 h compared to 2.60±0.51 h in T group. The total tramadol consumption in 24 h was significantly lower in E group with mean value of 193.33±17.59 mg compared with 233.33±25.82 mg in group T (p < 0.001) (Table 3).

**Table 3:** Comparison of quality of analgesia between two groups. SD= Standard Deviation, ^**^ highly significant, n= number of patients.

HRS	Group T (n=15)		Group E (n=15)		p-value
	Mean	±SD	Mean	±SD	
Time of administration of first analgesic ( Duration of analgesia)	2.60	±0.51	4.73	±0.7	<0.001**
Total Tramadol used in 24 h	243.33	±25.82	193.33	±17.59	<0.001**

There was significant (p < 0.001) difference in patient satisfaction score at 12 hours after surgery with mean value of 6.07±0.26 in group E compared to 3.40±0.91 in group T, however no significant difference in patient satisfaction was observed immediately after surgery between two study groups (Table 4). None of the patients in our study developed nausea and vomiting and no other opioid or bupivacaine-related side effects or any complication related to both block techniques were noted. None of the patients had any other significant side effects like bradycardia, hypertension, respiratory depression, itching or any drug related reactions in the postoperative period for which any intervention was required.

**Table 4:** Patient satisfaction score in both groups. n = number of patients, SD = standard deviation, ^** ^highly significant

Patient satisfaction score based on Likert scale	Group T (n=15)		Group E (n=15)		p- value
	Mean	±SD	Mean	±SD	
Immediately after surgery	5.80	±0.41	5.80	±0.41	0.500
12 h after surgery	3.40	±0.91	6.07	±0.26	<0.001**

## Discussion

With rising numbers of TAH, there are efforts to study various available options that aim to reduce postoperative pain in an attempt to hasten functional recovery and minimize the opioid-related systemic side effects. Regional nerve blockade is the favored alternative these days. In the present study, postoperative pain was assessed using a numerical rating scale (NRS), duration of analgesia, i.e., time of first analgesic demand postoperatively, a total analgesic requirement in 24 hours after surgery, and patient's satisfactory score. Hemodynamic variables and demographic profiles were recorded and compared. Patients were also assessed for any complications.

It was seen that NRS scores in the second, third, fourth, fifth, and sixth hours were significantly lower in the ESP group than in the TAP group. The results drawn in our analysis were in concurrence with studies done by Kamel et al. [8]. Similarly, Hamed et al. discovered that the VAS score in the ESP group was considerably lower for the first 12 hours postoperatively compared to the control group [9]. After laparoscopic cholecystectomy, When compared to oblique subcostal TAP, NRS values in the ESP group were significantly lower at postoperative 15, 30, and 60 minutes, as well as 12 and 24 h in a study done by Basak et al. [10]. VAS scores in the ESP group were considerably lower than in the TAP group at the second, fourth, sixth, eighth, and 12th postoperative hours and comparable at the 18th hour without any significant difference in patients who underwent sleeve gastrectomy, according to Abdelhamid et al. [11]. These findings were similar to the present study.

The demand for the first rescue analgesic was earlier in group T than in group E. Hamid et al. [9] found that the duration of analgesia was longer in the ESP group compared to the TAP group (p < 0.0001), it was consistent with the present study. Kamel et al. [8] observed that the demand for morphine postoperatively was delayed in the ESP group than in the TAP group (p < 0.0001). Similarly, Qi-hong et al. [12] observed that patients in the ESP block group had significantly lower sufentanil consumption compared with TAP block. Abdelhamid et al. [11] found that 24h postoperative cumulative pethidine consumption was higher in the control group compared to both erector spinae and transversus abdominis group (p < 0.001). However, when the erector spinae group was compared with the transversus abdominis group then pethidine consumption was less in the ESP group. Singh et al. [13] also observed that 24h morphine consumption was less in the group who received US-guided ESP block when compared with the control group (p = 0.01). Yildiz et al. [14] observed that the total amount of tramadol consumed in the first 24 hours was lower in Group E than in the control group (p < 0.01). Ciftci et al. [15] observed that postoperative fentanyl consumption was significantly lower in the ESPB group than in the sham block group (p = 0.009). Qi-hong et al. [12] conducted a study on a comparison of ultrasound-guided ESP block and oblique subcostal TAP who underwent laparoscopic colorectal surgery and observed the satisfaction score was higher in the ESP group (p < 0.01) which was similar to the current study. Kamel et al. [8] found that the TAP group had a statistically significantly higher number of unsatisfied patients than the ESP group (p = 0.03). No participants in this trial had nausea or vomiting, and there were no other opioid or bupivacaine-related adverse effects or complications associated with either block approach. None of the patients had any other significant side effects like bradycardia, hypertension, respiratory depression, itching, or any drug-related reactions in the postoperative period for which any intervention was required.

Limitations

Our limitation includes a short follow-up period so long-term effects could not be studied. We have used 30 ml of local anesthetic mixture but serum levels of LA were not checked. There is a limitation of clinical studies on ultrasound-guided ESP block which causes limited result comparisons. The sample size was small as less number of abdominal hysterectomies are performed under general anesthesia. Further larger studies are required to be conducted in order to detail the same for other abdominal and thoracic surgeries.

## Conclusions

The patients who received ESP block had reduced pain scores at all time intervals post-operative up to 24 h, which was statistically significant, hence proving the superiority of ESP block. The duration of analgesia in group E was longer than in group T as the time of first analgesic demand is significantly more in group E. Total dose of tramadol used in 24 h was significantly less in group E as compared to group T. Hence, we believe that bilateral ultrasound-guided ESP block provides more potent and long postoperative analgesia with less tramadol consumption compared to lateral TAP block after open total abdominal hysterectomy.
